# Local therapies for inflammatory eye disease in translation: past, present and future

**DOI:** 10.1186/1471-2415-13-39

**Published:** 2013-08-06

**Authors:** Shenzhen Tempest-Roe, Lavnish Joshi, Andrew D Dick, Simon RJ Taylor

**Affiliations:** 1 Faculty of Medicine, Imperial College London, London, UK; 2 Royal Surrey County Hospital NHS Foundation Trust, Guildford, Surrey, UK; 3 UCL Institute of Ophthalmology, 11-43 Bath Street, London, UK; 4 NIHR Biomedical Research Centre at Moorfields Eye Hospital NHS Foundation Trust and UCL Institute of Ophthalmology, Bristol, UK; 5 University of Bristol School of Clinical Sciences, Bristol, UK

## Abstract

Despite their side-effects and the advent of systemic immunosuppressives and biologics, the use of corticosteroids remains in the management of patients with uveitis, particularly when inflammation is associated with systemic disease or when bilateral ocular disease is present. The use of topical corticosteroids as local therapy for anterior uveitis is well-established, but periocular injections of corticosteroid can also be used to control mild or moderate intraocular inflammation. More recently, intraocular corticosteroids such as triamcinolone and steroid-loaded vitreal inserts and implants have been found to be effective, including in refractory cases. Additional benefits are noted when ocular inflammation is unilateral or asymmetric, when local therapy may preclude the need to increase the systemic medication.

Implants in particular have gained prominence with evidence of efficacy including both dexamethasone and fluocinolone loaded devices. However, an appealing avenue of research lies in the development of non-corticosteroid drugs in order to avoid the side-effects that limit the appeal of injected corticosteroids. Several existing drugs are being assessed, including anti-VEGF compounds such as ranibizumab and bevacizumab, anti-tumour necrosis factor alpha antibodies such as infliximab, as well as older cytotoxic medications such as methotrexate and cyclosporine, with varying degrees of success. Intravitreal sirolimus is currently undergoing phase 3 trials in uveitis and other inflammatory pathways have also been proposed as suitable therapeutic targets. Furthermore, the advent of biotechnology is seeing advances in generation of new therapeutic molecules such as high affinity binding peptides or modified high affinity or bivalent single chain Fab fragments, offering higher specificity and possibility of topical delivery.

## Introduction

Inflammatory eye disease encompasses a wide range of clinical phenotypes, and uveitis can be classified anatomically into either anterior, intermediate and posterior uveitis or panuveitis; and as acute or chronic disease, depending on whether it lasts more or less than 3 months in duration [1]. The Standardisation of Uveitis Nomenclature (SUN) criteria now form the standard for reporting uveitis clinical data [2]. The commonest type is acute anterior uveitis, in which 50% of people are HLA B27 positive, although they do not necessarily have an associated systemic disorder [3]. Chronic anterior uveitis lasts longer than 3 months and may or may not be associated with systemic disease. The rest of the disorders tend to be chronic, and the more serious types with posterior segment involvement have an increased incidence of visual loss, and approximately half of these patients have an associated systemic disease.

Corticosteroids remain the mainstay of treatment of all types of uveitis. Anterior uveitis is treated to control symptoms of pain, photophobia and redness, and to reduce complications such as posterior synechiae, cataract and macular oedema. Posterior segment inflammation usually requires treatment as it generates sight-threatening sequelae such as retinitis, macular oedema, optic disc oedema, chorioretinitis and retinal vasculitis. Topical corticosteroids are inadequate for this as they do not penetrate beyond the lens, so oral corticosteroids and second-line immunosuppressive agents are used, particularly in patients with an associated systemic disease and in those with bilateral ocular inflammation requiring treatment. Nevertheless, systemic administration is associated with significant side-effects, so there has been increasing interest in the local delivery of drugs to the eye and periocular tissues in order to avoid these complications.

This approach is not new. Traditionally, periocular injections of corticosteroids such as triamcinolone and methlyprednisolone have proved effective in controlling vitritis and mild to moderate macular edema in unilateral disease, but their use is limited by the need for repeat injections, IOP rises in corticosteroid responders and the induction of ptosis, orbital fat atrophy or orbital fat protrusion as a consequence of both the corticosteroid and the mode of injection [4,5]. More recently, intraocular delivery of corticosteroids has become widespread. Initially triamcinolone was used, but long-acting inserts are now becoming available, e.g. Retisert (Bausch & Lomb, Rochester, NY, USA) and Ozurdex (Allergan, Irvine, CA, USA).

Nevertheless, the local side-effects of corticosteroid delivery remain. Care also needs to be taken in cases of diagnostic uncertainty to ensure that there is not an infective cause for the uveitis, as this may be worsened by local therapy and depot corticosteroids can be difficult to remove, whereas oral corticosteroids can be rapidly stopped.

Owing to these side-effects, researchers have tried to move towards new non-corticosteroid alternatives. Some of these are old drugs, such as methotrexate, and others are based on the new so-called biological agents, in which monoclonal antibodies are directed against specific targets within the immune system, such as the anti-tumour necrosis factor (TNF)-alpha agents and the anti-vascular endothelial growth factor (VEGF) agents. This review article aims to outline agents currently in use for the local therapy of non-infectious uveitis, as well as those currently in translation from the laboratory to clinical use.

## Review

### Topical and subconjunctival therapy for ocular inflammatory disease

Topical corticosteroids have provided the mainstay of treatment for anterior uveitis since the 1950s, but do not penetrate far enough into the eye to control intermediate or posterior disease [6]. Their side-effects include cataract formation and raised intraocular pressure, in common with all corticosteroids, and are related to the strength of the corticosteroid and ocular penetration [6].

#### Corticosteroids

Dexamethasone sodium 0.1% and prednisolone acetate 1% are widely used and are broadly equivalent; difluprednate 0.05% has recently been introduced to the US and is considered more potent [7]. Rimexolone is a topical corticosteroid that was specifically engineered to generate less of an intraocular pressure rise by the elimination of a hydroxyl group. Randomised Controlled Trials (RCTs) suggest that it probably does induce less of a rise in intraocular pressure than either dexamethasone sodium 0.1% or prednisolone acetate 1% (the differences did not reach statistical significance [8]), but it is a weaker corticosteroid that is most useful in controlling chronic anterior uveitis in patients with established glaucoma or who are corticosteroid responders. Similarly, loteprednol etabonate 0.5% is associated with less of an intraocular pressure rise than prednisolone acetate 1% [9], but also has a reduced ability to control anterior chamber inflammation [9].

Subconjunctival corticosteroids in the form of dexamethasone or betamethasone may also be useful in the short-term treatment of severe anterior uveitis [10], and triamcinolone has recently been shown to be effective in the management of anterior scleritis without inducing necrotising disease, having a longer duration of effect than dexamethasone and betamethasone [11].

#### Non-steroidal anti-inflammatory drugs

Cyclooxygenase is a critical enzyme in the inflammatory process and catalyzes the biosynthesis of prostaglandins that disrupt the blood-ocular barrier, increase vasodilation, and facilitate leukocyte migration [12]. Non-steroidal anti-inflammatory drugs (NSAIDs) are potent inhibitors of cyclooxygenase enzymes and can be administered topically for the treatment of postoperative inflammation and macular oedema, either with or without concurrent corticosteroid administration [13].

#### Other agents

There are no other topical anti-inflammatory agents in widespread use for the treatment of uveitis. Topical Cyclosporine A was examined in several studies from the 1980s [14,15], but was shown to be ineffective in human disease. A study of the subconjunctival administration of sirolimus for intermediate or posterior uveitis was recently reported in which it was found to be safe and well tolerated (see Section “Anti-Tumour Necrosis Factor (TNF)-α agents” below) [16]; the results of a Phase 3 trial are awaited.

### Periocular therapy for ocular inflammatory disease

The numerous side-effects of oral corticosteroids are well-known and include gastric ulcers, weight gain, psychological disturbances, osteoporosis, diabetes, hypertension and suppression of growth in children [17]. In patients with unilateral or asymmetric disease, or in whom systemic administration of medication is less desirable, e.g. during pregnancy or in patients with a history of gastric ulceration, periocular injection can be useful to provide a depot of corticosteroid that successfully reaches the posterior segment to control inflammation [18-20].

#### Periocular corticosteroids

The precise mechanisms by which locally injected corticosteroids enter the eye are not known [21], but systemic drug levels remain low, and corticosteroids can be found in all layers of the eye, even at 30 days after a single subtenon injection of 40 mg of triamcinolone acetate, the highest levels being found in the choroid and retinal pigment epithelium [22]. Periocular corticosteroid injections can be administered either via the subtenon route or as an orbital floor injection [18]. Both procedures are safe, with a low risk of ocular penetration and of developing other side-effects of corticosteroid administration such as raised intraocular pressure and cataract [23-25]. The duration of effect is approximately 2 months [26].

#### Other periocular agents

Currently no other non-corticosteroid agents are injected periocularly for the treatment of ocular inflammatory disease.

### Intraocular corticosteroid therapy for ocular inflammatory disease

Intravitreal injections are now commonplace in ophthalmology, and are used for the treatment for a variety of ocular inflammatory and medical retinal disorders. The injection of dexamethasone into the vitreous had previously been used as an adjunct to vitrectomy, but it remains in the eye at therapeutic levels for hours only [27,28], and thus was superseded by triamcinolone as the intravitreal corticosteroid of choice. Triescence and Trivaris are triamcinolone preparations that are licenced for intraocular use in the US, but neither is available in the Europe, and Kenalog is commonly used off-label instead. However triamcinolone has a considerable side-effect profile in terms of cataract formation and raised intraocular pressure [29]. The injection-related side effects of intravitreal therapy resemble those of any other intraocular injection, and include endophthalmitis, intravitreal haemorrhage, rhegmatogenous retinal detachment, although these are rare. Other corticosteroid and non-corticosteroid agents have subsequently been developed, including sustained-release implants.

#### Intravitreal corticosteroid injections

Intravitreal injection of triamcinolone acetate (IVTA) in the treatment of uveitis is now commonplace, and involves the injection of corticosteroid directly into the vitreous body, thus achieving a higher concentration of intraocular corticosteroid [30]. The consensus for the recommended dosage of IVTA is 4mg, and the typical duration of effect is 3–4 months [31,32]. IVTA is most commonly used for the treatment of inflammatory cystoid macular oedema [33,34]. The systemic side-effects of IVTA are limited, as the triamcinolone is confined to the eye and serum levels have been shown not to be significant [35], but raised intraocular pressure is seen in 29-50% of patients within a year [33]. In most cases this can be controlled medically, but there have been a few reports of patients requiring surgical intervention [36]. Cataract development is also a common side-effect of IVTA in uveitic patients, and the rate of cataract progression is reported to be increased five-fold, particularly after multiple injections [29].

#### Short- and medium-acting intravitreal corticosteroid implants

The Surodex anterior segment delivery system (Oculex Pharmaceuticals, Sunnyvale, CA, USA) was designed to control inflammation after cataract surgery, and consists of a biodegradable device that is inserted into the anterior chamber and allows sustained release of corticosteroid over a period of seven days. It contains 60 μg dexamethasone incorporated into a polymer matrix of poly(lactic-glycolic)-acid, and achieves higher intraocular drug levels than conventional dexamethasone eye drops [37]. It is effective in the control of post-cataract surgery inflammation [37-39] but, as it only lasts for seven days, its usefulness does not extend beyond post-operative uveitis, and is not in widespread use.

The Ozurdex ‘bio-erodible’ dexamethasone implant is now licensed for the treatment of uveitis in the USA and Europe. This uses a solid polymer delivery system, in which biodegradable material is combined with dexamethasone to form a small rod-shaped implant which is injected into the vitreous using a specially designed injector. Dexamethasone is released over about 6 months, the pharmacokinetics demonstrating a high initial concentration peak in the vitreous followed by a longer period of low-level release before the implant dissolves completely to H_2_O and CO_2_, leaving no residue [40]. This has been shown to be effective in both adult [41] and paediatric [42] uveitis and it is hoped that it will cause less raised intraocular pressure and cataract than triamcinolone, but the phase III trial included only one implant and excluded steroid responders. Further data are awaited.

#### Long-acting intravitreal corticosteroid implants

Retisert is a long-term slow release intravitreal implant, which was based on those used to deliver ganciclovir to patients with cytomegalovirus retinitis, but which is smaller in size. The chosen corticosteroid is fluocinolone acetonide as it has high potency, low solubility and a very short duration of action in the systemic circulation, enabling the steroid pellet to be small, and reducing the risk of systemic side-effects. The implant is surgically placed into the vitreous cavity and pharmacokinetic studies in rabbits have demonstrated the delivery of constant levels of the corticosteroid to the posterior pole with no evidence of systemic absorption over approximately 2.5 years [43,44].

Its efficacy has been demonstrated in large studies [45], but it induces marked cataract formation such that all patients require cataract surgery within 3 years [45]. Significantly raised intraocular pressure is also very common, with up to 40% of patients requiring trabeculectomy surgery [45]. Additional side-effects include scleral thinning over the implant in some cases, vitreous band formation, and the development of cytomegalovirus retinitis and endotheliitis [46]. In view of its effects on intraocular pressure and cataract, it is worth noting that insertion of the implant can be combined safely with glaucoma drainage device placement [47] or with phacoemulsification and intraocular lens insertion [48].

The Multicenter Uveitis Steroid Treatment (MUST) trial compare local control of intermediate and posterior uveitis with Retisert implants (implanted bilaterally for bilateral disease) with aggressive oral systemic therapy, and found no significant differences between each treatment arm at two years in terms of vision, although macular oedema did appear to be better controlled in the implant arm [49]. The trial has recently been extended for a further four years to see whether significant differences emerge over a longer timespan.

#### Other intravitreal corticosteroid inserts

Iluvien (Alimera Sciences, Alpharetta, GA, USA) is another fluocinolone acetonide intravitreal insert which is designed to deliver corticosteroid to the retina for up to three years as a treatment for diabetic macular oedema [50]. It uses the same drug matrix as Retisert, but is thought to release a lower dose of drug (0.2 μg/day or 0.5 μg/day) than Retisert (nominally 0.59 μg/day) [50], and is injected through a proprietary 25-gauge injector system in an outpatient setting, in contrast to the surgical setting required to implant the Retisert device. Phase II studies in diabetic macular oedema suggest the possibility of a lower rate of IOP rise than with Retisert, although the numbers in the study were relatively small, the follow-up period short, and there was still significant incidence of cataract progression, suggesting the occurrence of significant corticosteroid side-effects [51]. There is no published evidence for its use in uveitis. The I-Vation implant (SurModics, Eden Prairie, MN, USA) is based around triamcinolone, and is reported to have a duration of release of over a year. It has a unique implantation mechanism in which a screw-shaped device is twisted through the pars plana, although little published data is currently available on its use [50,52].

### Intravitreal non-corticosteroid therapy for ocular inflammatory disease

In an attempt to avoid the ocular side-effects of intraocular corticosteroids, attention has been focused on the development of other agents. This has involved the trial of both established immunosuppressive agents and also the novel so-called biological therapies, with varying degrees of success.

#### Non-steroidal anti-inflammatory drugs

Ketorolac does not reach significant levels in the vitreous or retina after topical or systemic application [12,53]. A recent prospective phase I trial of intravitreal ketorolac in ten patients showed some effect in treating intraocular inflammation and macular oedema [54]. However, a similar pilot study of intravitreal diclofenac showed no benefit [55].

#### Conventional immunosuppressive agents

Intraocular methotrexate has long been used to treat intraocular lymphomas associated with primary central nervous system lymphoma [56,57], but has more recently been tried in uveitis [58,59]. In one prospective study it was found to be effective in reducing vitritis and macular oedema without raising the IOP in patients with a history of steroid response. Interestingly, the onset of effect was within one week and lasted approximately three months, with no statistical difference between the best visual acuity obtained after methotrexate injection and after previous corticosteroid treatment, including IVTA injection [59]. A larger collaborative series is currently in press and has suggested that this drug may induce longer-term remission in some patients [60,61].

There is some published evidence of the successful use of cyclosporine-loaded poly(lactic co-glycolic) spheres in animal models [62], but this has not been successfully translated into the treatment of human disease. Intravitreal tacrolimus has shared a similar fate [63,64].

#### Anti-vascular endothelial growth factor (VEGF) agents

VEGF inhibition has also been tried as a non-corticosteroid intraocular treatment for uveitic CME, owing to its induction in inflammation, its role in increasing vascular permeability and the finding of increased levels in eyes with uveitic macular oedema [65-69]. Several small retrospective studies have been reported using both bevacizumab and ranibizumab, although bevacizumab is more commonly used owing to its lower cost. Intravitreal bevacizumab does appear effective in reducing central macular thickness, but the studies do not generally suggest a statistically significant increase in visual acuity [65,70,71]. Similar results have been achieved with ranibizumab [72].

Interestingly, three studies have compared the use of anti-VEGF treatment to IVTA in the treatment of uveitis-associated CME. Two retrospective reports comprising 31 eyes treated with IVTA and 26 eyes treated with intravitreal bevacizumab indicated a trend for better visual improvement and decreased macular thickness in IVTA-treated eyes [73,74]. A further prospective report with longer follow-up indicated an improved visual outcome for eyes treated with IVTA, once the confounding effect of cataract had been removed [75].

Similar to methotrexate, anti-VEGF agents have the advantage over IVTA of being much less likely to cause cataract progression or a rise in IOP. However, they have less anti-inflammatory effect, making them less suitable for the treatment of CME primarily driven by inflammation [76] or if there is extensive breakdown of the blood-retina barrier [65]. The risk of serious cardiovascular events also remains controversial [77,78].

#### Anti-tumour necrosis factor (TNF)-α agents

Local inhibition of TNF-α is a new and promising therapeutic direction, but clinical trials in patients have had mixed results. The initial rationale for injecting anti-TNF-α drugs intraocularly was their efficacy when used systemically, even though their purported mechanism of action may not be effective when delivered locally [79-82]. Infliximab is a humanized, chimeric monoclonal antibody directed against TNF-α and clinical experience with its intravitreal use in uveitis is limited to one small pilot study of 10 eyes with chronic non-infectious uveitis that were unresponsive to systemic steroids [83]. Intravitreal injection of 1.5 mg infliximab led to significant improvements in best corrected visual acuity, with a significant decrease in the central macular thickness and vitreous haze grading, but the follow-up period in this study was very short at four weeks. However, in one study in which 0.5 mg infliximab was injected for either diabetic macular oedema or neovascular age related macular degeneration, intraocular inflammation developed in three of four treated eyes [84], and in another study in which eyes with refractory diabetic macular oedema were treated with 1–2 mg infliximab, 42% of eyes receiving the 2mg dose developed severe uveitis, most of which subsequently required pars plana vitrectomy [85]. Not surprisingly, there has been a call for a moratorium on the clinical use of intravitreal infliximab outside of well-designed trials [86].

Adalimumab, a humanized monoclonal antibody against the soluble and membrane-bound TNF has recently been considered for intravitreal injection. Evidence of safety has been reported in rabbits [87], but clinical use in uveitic CME is limited to one small study of 8 eyes that were unresponsive to treatment with intraocular steroids and anti-VEGF injection. While there were no safety concerns with a dose of 1 mg, it failed to produce any significant improvement in vision or reduction of macular thickness [88]. Pre-clinical studies of the TNF inhibitor ESBA105 have also suggested good intravitreal and neuroretinal bioavailability [89], and it may be that newer agents can overcome the problems seen with the older chimeric antibody infliximab.

#### Sirolimus

Sirolimus, also known as rapamycin, was isolated in the 1970s from *Streptomyces hygroscopicus* in soil samples from Easter Island [90]. It is an immunosuppressant that works through inhibition of the mammalian target of rapamycin (mTOR) by binding to the immunophilin FK protein 12 (FKBP-12), and thus interrupts the inflammatory cascade that leads to T-cell activation and proliferation. It also suppresses T-cell proliferation through the inhibition of IL-2, IL-4, and IL-15 via both calcium-dependent and calcium-independent pathways [91]. A study of intravitreal and subconjunctival administration of sirolimus in 30 patients was recently reported in which it was reported to be safe and tolerable [16]. The results of a Phase III trial are currently awaited.

### Future directions

Local treatment remains an attractive therapeutic option for uveitis, as it has the potential for avoiding systemic adverse events. Research continues into developing novel corticosteroids that maintain their anti-inflammatory effects whilst having an improved ocular side-effect profile, but the greatest hope must lie in the development of non-corticosteroid therapeutic options. In support of this goal, understanding of the pathophysiology of uveitis has advanced over the past decade, and mechanisms of ocular damage are increasingly being understood [92,93].

This is particularly true in the broad context of Matzinger’s danger hypothesis, which helps to explain the ways in which the immune system is designed to detect self from non-self [94]. Non-infectious uveitis is generally understood as representing an autoimmune phenomenon, but experimental non-infectious uveitis requires initial activation of innate immunity prior to the generation of specific T cell responses, and there is good evidence to suggest that this also applies in human disease [92]. This presents a therapeutic opportunity as autoimmunity and autoinflammation evoke different molecular pathways that may generate different potential molecular targets. For example, improved understanding of how danger is sensed by the immune system, and how inflammasomes are subsequently involved in the activation of caspase-1 to release IL-1β and IL-18 [95] leads to the potential for anti-IL-1 or anti-caspase-1 therapies in some uveitic conditions [96]. Short interfering RNA (siRNA) approaches may also offer a mechanism by which to target caspases, and caspase-2 has already been targeted successfully in an animal model of retinal ganglion cell loss [97]. Similarly, it may be possible to block pro-inflammatory cytokines such as IL-1β and IL-6 locally [89], or to target upstream so-called danger sensors, such as the purinergic ATP receptors [98], or even cell function regulators such as the sirtuins [99].

Other promising strategies include preventing cells from entering the target organ by inhibition of either adhesion or migration through endothelium (anti-α4-integrin (natalizumab)) [100], or via preventing efflux from lymph nodes by blocking sphingosine-1-phosphate receptor (fingolimod) [101], or targeting other effector cells, such as macrophages, through complement inhibition or stimulation of the CD200 macrophage inhibitory receptor. Anti-CD20 (rituximab) has also shown efficacy when given systemically for orbital inflammation [102,103], although it has not been used intravitreally other than for lymphoma [104], and the efficacy of some of these treatments is helping to illuminate novel mechanistic pathways and challenge previous understanding of disease pathophysiology.

Furthermore, the advent of biotechnology is seeing advances in generation of new therapeutic molecules such as high affinity binding peptides or modified high affinity or bivalent single chain Fab fragments, offering higher specificity and possibility of topical delivery. For instance, the TNF-α inhibitory single-chain antibody fragment ESBA105 has been shown to reach therapeutic levels in all ocular compartments following topical administration in rabbits [105], and it is hoped that it will be possible to translate similar technology into clinical use. Indeed, the ability to deliver effective uveitis therapy to the posterior segment through the topical route truly would provide a step change to the advantages of local therapy.

## Conclusions

In conclusion, the eye offers both a unique window on the functioning of the immune system and a unique opportunity for the effective local treatment of autoimmune and autoinflammatory disorders. Given the plethora of potential mechanisms and associated targets that increased understanding of its pathophysiology has introduced, it seems unlikely that the long-term future of therapy lies in corticosteroids, but instead that this increased knowledge will offer the opportunity for effective targeted treatment of the molecular mechanisms underlying ocular inflammation whilst minimising local and systemic side-effects.

## Competing interests

ST has received consultancy fees from Allergan Inc. and Novartis Inc. This work has not previously been presented or reported in any form.

## Authors’ contributions

ST-R, LJ and ST drafted the article, ST and AD edited the article. All authors read and approved the final manuscript.

## Pre-publication history

The pre-publication history for this paper can be accessed here:

http://www.biomedcentral.com/1471-2415/13/39/prepub
